# Identification of Mosquito Bloodmeals Collected in Diverse Habitats in Malaysian Borneo Using COI Barcoding

**DOI:** 10.3390/tropicalmed5020051

**Published:** 2020-04-01

**Authors:** Katherine I. Young, Joseph T. Medwid, Sasha R. Azar, Robert M. Huff, Hannah Drumm, Lark L. Coffey, R. Jason Pitts, Michaela Buenemann, Nikos Vasilakis, David Perera, Kathryn A. Hanley

**Affiliations:** 1 Department of Biology, New Mexico State University, Las Cruces NM 88003, USA; jtmedwid@nmsu.edu (J.T.M.); khanley@nmsu.edu (K.A.H.); 2 Department of Pathology, University of Texas Medical Branch, Galveston, TX 77555, USA; srazar@utmb.edu (S.R.A.); nivasila@utmb.edu (N.V.); 3 Department of Microbiology and Immunology, University of Texas Medical Branch, Galveston, TX 77555, USA; 4 Institute for Translational Sciences, University of Texas Medical Branch, Galveston, TX 77555, USA; 5 Department of Biology, Baylor University, Waco, TX 76706, USA; Robert_Huff1@baylor.edu (R.M.H.); Jason_Pitts@baylor.edu (R.J.P.); 6 School of Veterinary Medicine, University of California Davis, Davis, CA 95616, USA; hdrumm@ucdavis.edu (H.D.); lcoffey@ucdavis.edu (L.L.C.); 7 Department of Pathology, Microbiology & Immunology, University of California Davis, Davis, CA 95616, USA; 8 Department of Geography, New Mexico State University, Las Cruces, NM 88003, USA; elabuen@nmsu.edu; 9 Center for Biodefense and Emerging Infectious Diseases, University of Texas Medical Branch, Galveston, TX 77555, USA; 10 Center for Tropical Diseases, University of Texas Medical Branch, Galveston, TX 77555, USA; 11 Institute for Human Infection and Immunity, University of Texas Medical Branch, Galveston, TX 77555, USA; 12 Institute of Health and Communiti Medicine, Universiti of Malaysia Sarawak, Sarawak 94300, Malaysia; dperera@unimas.my

**Keywords:** mosquito, vector, host, bloodmeal, arbovirus, Borneo, land cover and land use change, *Aedes*, dengue virus

## Abstract

Land cover and land use change (LCLUC) acts as a catalyst for spillover of arthropod-borne pathogens into novel hosts by shifting host and vector diversity, abundance, and distribution, ultimately reshaping host–vector interactions. Identification of bloodmeals from wild-caught mosquitoes provides insight into host utilization of particular species in particular land cover types, and hence their potential role in pathogen maintenance and spillover. Here, we collected 134 blood-engorged mosquitoes comprising 10 taxa across 9 land cover types in Sarawak, Malaysian Borneo, a region experiencing intense LCLUC and concomitant spillover of arthropod-borne pathogens. Host sources of blood were successfully identified for 116 (87%) mosquitoes using cytochrome oxidase subunit I (COI) barcoding. A diverse range of hosts were identified, including reptiles, amphibians, birds, and mammals. Sixteen engorged *Aedes albopictus*, a major vector of dengue virus, were collected from seven land cover types and found to feed exclusively on humans (73%) and boar (27%). *Culex tritaeniohynchus* (n = 2), *Cx. gelidus* (n = 3), and *Cx. quiquefasciatus* (n = 3), vectors of Japanese encephalitis virus, fed on humans and pigs in the rural built-up land cover, creating potential transmission networks between these species. Our data support the use of COI barcoding to characterize mosquito–host networks in a biodiversity hotspot.

## 1. Introduction

Mosquito-borne viruses such as dengue virus (DENV), yellow fever virus (YFV), Zika virus (ZIKV), and chikungunya virus (CHIKV) have gained notoriety for causing explosive, global pandemics sustained in transmission between humans and the urban-living mosquitoes *Ae. aegypti* and *Ae. albopictus* [1,2,3]. However, maintenance of such human-endemic cycles are actually quite rare among mosquito-borne viruses, the majority of which are zoonotic [4,5,6]. In general, mosquito-borne viruses are maintained in one of three cycles defined by specific hosts, vectors, and land cover types: 1) enzootic cycles involve wildlife hosts and vectors in natural environments, 2) rural epizootic cycles involve domestic animals and vectors in agriculture or rangelands, and 3) urban cycles involve humans or urbanized wildlife as hosts and urbanized vectors found in cities or suburbs [4]. Although distinct, these transmission cycles can be linked by spillover, the transmission of an arbovirus from a reservoir host via a bridge vector to a novel host [1,6,7]. For example, DENV, CHIKV, YFV, and ZIKV all arose in ancestral, enzootic cycles and spilled over to establish human-endemic cycles. Spillover can sometimes require amplification, which is infection of transmission-competent hosts that are fed upon by vectors that bridge to an established cycle and to novel, dead-end hosts from which no further transmission occurs [4,5]. Japanese encephalitis virus (JEV) is the main cause of human viral encephalitis in Asia but is maintained in an enzootic transmission cycle between mosquitoes and wading birds. However, the *Culex* mosquitoes that act as vectors are generalists and their feeding habits enable transmission to pigs. In pigs, the virus can amplify to high titers, and subsequently transmit to other hosts, including humans who are dead-end hosts for this virus [8].

The ability of a given vector species to mediate spillover depends on the breadth of its host utilization and its distribution across different habitats. Mosquitoes that are host and habitat generalists are more likely to act as bridge vectors than are highly specialized mosquitoes [9,10,11,12]. A number of extrinsic and intrinsic factors influence the host preference of mosquitoes, including olfaction, physiology, genetics, climate, host body heat, host defensive behavior, and host density [12]. However, these traits are complex, and determining innate host preferences for mosquitoes experimentally via lab or field studies can be biased by the use of colonized mosquitoes, seasonal and circadian changes in host and vector density, and variation between mosquito populations, among other factors [9,12]. An alternative to the experimental approach is to collect blood-engorged mosquitoes from the wild and use molecular methods to identify host use. While bloodmeal identification may not provide a complete picture of host preference [9], it can provide an accurate representation of host utilization patterns within a specific location and timeframe. 

The current study used bloodmeal analysis to characterize mosquito–host interactions in Malaysian Borneo, a biodiversity hotspot [13] that is experiencing extreme rates of land cover and land use change (LCLUC) [14,15,16,17]. Since 1980, there has been steady clearance of native forests in Borneo, including old-growth dipterocarp, peat swamp, heath, and mangrove forests, primarily for logging and conversion to large plantations [14,15,16,17]. Meanwhile, high-density urban areas have expanded and people in low-density rural regions continue to clear forested areas for subsistence farming [18]. LCLUC plays an important role in arbovirus transmission by reshaping vector–host interactions via effects on vector and host distributions, abundance, and diversity [6,19,20,21,22]. In Borneo, extensive spillover of arboviruses [23,24,25] and other mosquito-borne pathogens [26,27] has been documented in the past several decades. Of particular concern are a number of cases of spillover of sylvatic DENV into humans [28,29,30,31], as each spillover event has the potential to launch a new strain of this virus into human-endemic transmission.

Here, we opportunistically sampled bloodfed mosquitoes from mosquito collections in nine land cover types, ranging from primary forest to barren land, in Sarawak Malaysian Borneo in 2016 and 2017. From these samples we sought: 1) To identify mosquito bloodmeal sources, using cytochrome oxidase subunit I (COI) barcoding, and 2) to represent the detected host–vector networks across these nine land cover types, while acknowledging that, given the number of samples analyzed, this represents only a small subset of the complete network within each land cover.

## 2. Materials and Methods

### 2.1. Study Site and Design

**Figure 1:**
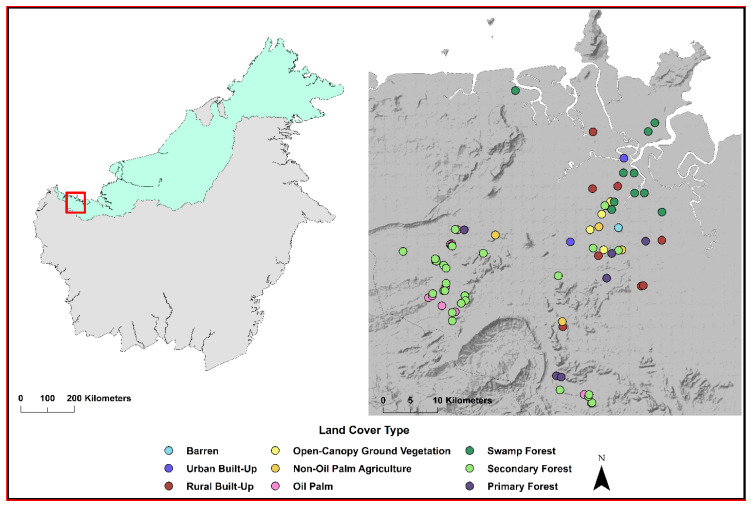
Study location in Sarawak, Malaysian Borneo is shown by the red box in the left panel. Sites where a bloodmeal was collected and the host was identified are shown in the right panel. The land cover type of each site sampled is designated by color.

The study represents a concatenation of three projects focused on mosquito collections within the Kuching, Samarahan, and Serian administrative divisions in Sarawak, Malaysian Borneo (central coordinates: 1.5533° N, 110.3592° E), between April 2016 and June 2017 (Figure 1). Sarawak is considered Tropical Rainforest (Köppen-Geiger classification [32]) and receives rainfall year-round. This region of Sarawak comprises a mosaic of land covers including developed cities, peri-urban to rural villages, expansive cash crop plantations, small subsistence farms, and primary and secondary forests.

The first project, termed the KK project, was a resampling of 15 sites across three land cover types in the village of Kampung Karu in Sarawak from April to May of 2016. Mosquito sampling was focused around homesteads, which are designated as rural built-up sites in this study, mixed agricultural fields, and secondary forests surrounding the village. The sampling method is thoroughly described by Young et al. (2017) [33]. Briefly, five sites in each land cover type were selected in the field based on observations made between 2013 and 2014 [33]. In 2016, these sites were resampled to measure changes in the abundance and diversity of mosquitoes and to evaluate land cover change between 2013/2014 and 2016 sampling years [33]. Thus, a total of 15 sites were sampled for this project, of which two yielded at least one blood-engorged mosquito for a total of two mosquitoes from this project.

The second project, called the OP project, began in June 2016 and ended in January 2017. Mosquito sampling was undertaken to assess the effects of oil palm insertion into forests on mosquito diversity, abundance, and distributions (Young et al. (2019) in revision for Ecosphere). Sampling was conducted in oil palm plantations, forests conterminous with those plantations, and the edge between these two land cover types. Google Earth satellite imagery from 25 July 2015 and 2 October 2015 was used to digitize >15 hectare oil palm plantations and adjacent forest boundaries in ArcGIS version 10.3.1 (ESRI, Redlands, California). Thirty-four sampling sites were randomly distributed at each of 10 isodistances, bands of equal distances measured from the edge of oil palm plantations and adjacent forests, within the boundaries of these land cover types; interior plantations to forest edges (–10 m, –50 m, –20 m, and –100 m), edge (0 m), and edge to the interior of contiguous forests (+10, +20, +50, +100, +500 m). A total of 340 sites were sampled for this project, of which 36 yielded at least one blood-engorged mosquito for a total of 43 mosquitoes from this project.

The third project, called the LC project, was undertaken from February to June 2017. Adult mosquito sampling was used to describe patterns in mosquito diversity, abundance, and distribution associated with different land cover types in Sarawak. Landsat 8 Operational Land Imager (OLI) imagery covering the study area from 13 October 2016, was downloaded via USGS EarthExplorer (Landsat 8 imagery courtesy of U.S. Geological Survey; https://earthexplorer.usgs.gov) and atmospherically and topographically corrected using ATCOR 3 [34,35](Atmospheric Corrections Software). Spectral and spatial features derived from the 8 multispectral OLI bands as well as topographic features derived from the Shuttle Radar Topography Mission (SRTM) Digital Elevation Model (DEM) [36,37] were combined in a 577-band layer stack. This layer stack was then classified using Random Forest and 600 Google Earth-based training/testing sites into eleven major land cover types: Urban built-up land, rural built-up land, agricultural land, primary forest, secondary forest, swamp forest, mangrove forest, open-canopy ground vegetation, barren land, and water. The overall classification accuracy was 80.2%. We subsequently generated 50 random points in each of eight of the land cover types; barren land, urban built-up land, rural built-up land, open-canopy ground vegetation, agricultural land, swamp forest, secondary forest, and primary forest. In total 132 sites were sampled from these eight land cover types, of which 36 yielded at least one blood-engorged mosquito for a total of 89 mosquitoes from this project.

At the completion of all projects, a total of nine land cover types were sampled; barren land, urban built-up land, rural built-up land, open-canopy ground vegetation, non-oil palm agricultural land, oil palm plantation, swamp forest, secondary forest, and primary forest; all are described in detail in Table S1 and Figure S1. Criteria for land cover designation, described in Table S1, was consistent across projects. 

### 2.2. Mosquito Collection and Identification of Bloodfed Females

For the KK and OP projects, adult mosquitoes were collected using a backpack aspirator (Bioquip, Rancho Dominguez, CA, USA) [33], while for the LC project mosquitoes were collected at each site using a combination of gravid traps (Bioquip), CO_2_ baited light traps (Clarke, St. Charles, IL, USA), BG sentinel traps (Bioquip), and aspiration via a Prokopac aspirator. Aspiration for the KK project was conducted for a duration of five minutes at nine trapping locations within each site and focused on open air, vegetation, and the body of the sampler [33]. For the OP and LC projects, aspiration was conducted around a 5 m radius from the center of each site by moving the aspirator in sweeping motions through the open air and directly onto or near ground vegetation and taller vegetation up to chest height, including trees and shrubs. For traps, one of each trap type was placed 5 m apart within the site and run until retrieval the following day. All BG sentinel traps were used with an octenol scent-bait (Bioquip). Gravid traps were placed over dark colored bins filled with hay infused water at a ratio of 3.8 g of hay: 1L water. Light traps were suspended at head height from either vegetation or existing structures and supplied with a yeast CO_2_ bait made from 500 mL water, 7g INS yeast, and 50 g of coarse sugar, all acquired from a local grocery store [38].

Captured mosquitoes from all projects were transported in the collection containers used for each trap type. The LC project transported trap containers in a cooler with ice packs back to the Universiti of Malaysia Sarawak, where mosquitoes were killed by placing them at –20 °C, but the KK and OP projects transported trap containers directly without ice packs. Mosquitoes were then morphologically identified to the lowest taxonomic level possible using available keys [39,40,41] and the Walter Reed Biosystematics Unit system [42]. Female mosquitoes with visibly engorged abdomens were identified and their abdomens were separated and stored in individual tubes at –80°C for bloodmeal analysis. The LC project retained individual mosquito legs in a separate tube from all bloodfed females to be used for molecular identification of mosquitoes if needed; however, the KK and OP projects did not retain legs for this purpose. 

### 2.3. Bloodmeal DNA Extraction and COI Amplification

DNA was extracted from the abdomen of blood-engorged mosquitoes according to a protocol described by Thiemann et al. (2012) using the Qiagen DNeasy Blood and Tissue Kit (Qiagen, Germantown, MD, USA) [43]. Twenty µl of proteinase K was added to each tube and a sterile pestle (Fisher Scientific, Hampton, NH) was used to gently crush the abdomen against the side releasing the bloodmeal. Next, 120 µl Buffer ATL was added and samples were incubated over night at 56 °C. Column extraction was then completed according to the manufacturer’s instructions (Qiagen, Germantown, MD, USA).

Amplification of the cytochrome oxidase subunit I (COI) gene was conducted following one of two protocols. First, a nested PCR protocol described by Thiemann et al. (2012) was used on extracted DNA [43]. Briefly, the entire COI gene was amplified using primers that anneal to the tRNA regions flanking the gene at the 5’ and 3’ termini [43]. Four primer pairs were included in each reaction to cover a wide taxonomic breadth of hosts (Table S2) [43]. The PCR reaction was performed using Amplitaq (Applied Biosystems, Foster City, CA), and the volume of other reagents used are described in Table S2 under the tRNA protocol. Next an approximately 700 base pair fragment of the COI gene was amplified using HotStarTaq (Qiagen, Hilden, Germany) from each of the tRNA products using 6 internal primers: 3 forward and 3 reverse (Table S2) [43]. These forward and reverse primers were mixed in 1:1:2 ratios: VFmix is a 1:1:2 ratio of VF1, VF1d, VF1i, and VRmix is a 1:1:2 ratio of VR1, VR1d, VR1i (Table S3). All bloodmeal samples were screened using this protocol, and those that failed to generate products were then subject to a COI protocol described by Townzen et al. (2008) [44]. In brief, a 324 base pair region of COI was amplified from the extracted DNA using primers designed for a broad range of vertebrates and a recombinant Taq DNA polymerase (Invitrogen, Carlsbad, CA) (Table S2, Table S3). All PCR reactions were visualized on a 1.5% TAE agarose gel and included both negative and positive controls; the latter included DNA extracted from the blood of one amphibian (*Anaxyrus boreas*) (gift of Dr. Jamie Voyles, University of Nevada Reno IACUC 00698), one bird (*Melopsittacus undulates)* (gift of Dr. Timothy Wright, New Mexico State University (NMSU) IACUC 2016-006), one mammal (*Ovies aries*) (gift of Dr. Ryan Ashley, NMSU IACUC 2017-020), and one reptile (*Acrantophis spp.*) (gift of the Alamagordo Zoo, Alamagordo, New Mexico) species.

### 2.4. Sequence Analysis and Host Identification

Sanger sequencing was performed on samples with PCR products of the expected amplicon size. For samples amplified using the Thiemann protocol [43], the VFli primer was used for sequencing. Samples amplified using the Townzen protocol [44] were sequenced using both forward and reverse primers. Returned sequences were trimmed and, if amplified using the Townzen protocol, forward and reverse reads were aligned using ClustalW in Geneious software (Newark, NJ). Alignments were used to generate a consensus sequences for these samples. All samples were BLAST-searched against BOLD and NCBI databases. Generally, sequences with less than 3% sequence divergence are considered conspecific in DNA barcoding [45,46]. However, this designation is arbitrary, and it can be appropriate to use more broad sequence divergence criteria [47]. For host identification, sequences with ≥95% sequence identity were considered conspecific so long as the host identified was an endemic species of Borneo and frequently found in the land cover detected. Sequences with ≥80% identity were considered congeneric and between 77%–80% identity confamilic. However, if sequences had ≥80% identity to a species that is not known to occur in Borneo, or the surrounding region, they were considered confamilic. 

### 2.5. Molecular Confirmation of Bloodfed Mosquito Species

To confirm morphological identifications of bloodfed mosquitoes, we attempted COI barcoding using a protocol described by Young et al. (2017) [33] from the LC project leg tissues. Briefly, total DNA and RNA was extracted using Trizol (Invitrogen, Pittsburgh, PA). Primers described by Folmer et al. (1998) [48] were then used in a PCR reaction to amplify a 710 base pair region of the COI gene. If the PCR reaction failed to yield a product, RNA was used in an RT-PCR reaction with the same primers to create cDNA for this region of the COI gene. The resulting products were then sequenced using both the forward and reverse primers, consensus sequences were generated, and resulting sequences were BLAST-searched against the BOLD and NCBI databases (Young et al. 2017) (Supplementary file SF1).

### 2.6. COI Cloning and Molecular Identification of Mosquitos from Bloodmeal DNA

Using the methods described in Section 2.5 in combination with morphological identification, three mosquitoes in which a bloodmeal had been amplified could be confidently identified to the level of species, two mosquitoes to genus, and one to family. To attempt to achieve more species-level identifications, we subjected a subset of 20 samples to cloning prior to sequencing. First, a PCR reaction was performed using primers that amplify an approximately 250 bp region of mosquito COI (Table S2) and 2x GoTaq Green PCR Master mix (Promega, Madison, WI) (Table S4). After PCR amplification, cloning using 2 µl of each reaction product was performed with the TOPO TA cloning kit (ThermoFisher Scientific, Waltham, MA) according to the manufacturer’s protocol. Colonies were picked and placed into 3 mL of LB+Amp (100 ug/mL) liquid media and incubated at 37 °C for 16 h with shaking at 220 rpm. Miniprep liquid cultures (GeneJET, ThermoFisher Scientific, Waltham, MA) were created, diluted to 100 ng/µl, and then sequences at the UT Austin Sequencing Facility. These sequences were next compared to the BOLD and NCBI databases to confirm mosquito species. Because this amplicon was shorter than the traditional, 500-bp barcodes generated above, many of these sequences provided lower-level resolution. If a sample had been identified to species a priori using morphology and the sequence of the cloned amplicon had ≥98% identity to that species, we considered our morphological ID to be confirmed. If we had been unable to pinpoint a species-level ID and the sequence had equal percent identity across multiple species within the same genus, we considered this genus the lowest level of taxonomy achieved. If our sequence had equal percent identity across multiple genera, it was considered unresolved and the morphological identification was used as the final identification. 

### 2.7. Descriptions of Host and Vector Networks

Due to the small number of bloodfed mosquitoes collected, we did not attempt statistical analyses of these data. To visualize host and vector networks, associations between vectors and hosts detected from bloodmeal identification, alluvial plots were created for each land cover type from which >5 bloodmeals were identified. Alluvial plots show the frequency of connections between categorical groups. The nodes of the plot, i.e., the black bars on the left and right sides, represent a category of interest, mosquito and host taxa in this case. The size of the node is relative to the proportion that each category represents of total samples. The flows of the plot, colored fans in the center of each plot, represent the connections between nodes and the size of each flow is proportional to the frequency at which those connections are made. Alluvial plots were created using RAWGraphics [49].

## 3. Results

### 3.1. Identification of Mosquito Bloodmeal Sources

Of 7299 mosquitoes collected from all projects, 134 were blood-engorged females, representing <2% of the total mosquitoes sampled. The relatively sparse yield of bloodfed mosquitoes is typical of studies in natural, tropical habitats [50,51,52,53,54,55]. For example, Brown et al. collected 127 bloodfed mosquitoes using three trap types in Sabah, Malaysian Borneo and were able to identify host species in 30% of these [52]. The small total number of blood fed mosquitoes in this study and the variation in sample size across land cover types, ranging from 1 mosquito in barren land to 42 mosquitoes in secondary forests, precluded statistical analysis of arbovirus spillover risk to humans.

The 134 specimens analyzed included eight genera of mosquitoes: *Aedes, Anopheles, Armigeres, Culex, Mansonia, Topomyia, Tripteroides, Uranotaenia*; one group identified to the Aedini tribe; and one specimen only identified to the Culicidae family. Engorged mosquitoes were collected from 74 sites in 9 land cover types in Sarawak. Four of these land cover types, barren, urban, open canopy ground vegetation and swamp forest, were sampled only in the LC project. Oil palm agriculture was sampled only in the OP project. Rural built-up (LC = 8 sites, KK = 1 site), non-oil palm agriculture (LC = 3 sites, KK = 1 site), secondary forest (OP = 24 sites, LC = 5 sites), and primary forests (LC = 3 sites, OP = 3 site) were sampled in two separate projects. However, since land cover classifications were consistent across projects, this is unlikely to confound consideration of networks within a land cover. Of the sampling methods used, 76% of the bloodfed mosquitoes were captured via aspiration, 19% via gravid traps, 4% via BG Sentinel traps, and 1% via light traps. Of the 134 blood fed mosquitoes, hosts were identified from 87% (n = 116). 

Twenty-one unique host families were detected, including amphibians: Dicroglossidae Megophryidae, Ranidae; birds: Columbidae, Fringillidae, Phasianidae, Pittidae, Remizidae, Strigidae, Timaliidae; mammals: Canidae, Cervidae, Cynocephalidae, Hominidae, Muridae, Suidae, Tarsiidae, Tragulidae, and reptiles: Agamidae, Scincidae, Varanidae. Host identity was resolved to species for 92 specimens, to genus for 15, and to family for nine. No evidence of multiple hosts was detected in any mosquito.

With 62 specimens, *Culex* mosquitoes made up the majority of the collection and also encompassed the widest range of hosts including, reptilian (15%), amphibian (1%), avian (32%), and mammalian (52%) taxa. Similarly, a wide range of hosts were detected from 16 *Uranotaenia* specimens, with over half coming from reptilian and amphibian hosts (81%), 13% from avian hosts, and 6% from mammalian hosts. Humans were the only hosts detected from 16 mosquitoes identified only to the Aedini tribe. All sixteen *Aedes* samples collected were identified as *Ae. albopictus*. Seventy-five percent of *Ae. albopictus* bloodmeals identified were humans, and the remaining from *Sus scrofa* (wild boar) and *Sus barbatus* (bearded pig). Only a single sample each was collected from the remaining mosquito genera and reptiles and mammals were detected as hosts for these (Table 1, Supplementary file SF1).

### 3.2. Identification of Mosquitoes Collected

Molecular taxonomy using COI barcoding was attempted on 104 of the bloodfed mosquito samples from which hosts had been identified. Eighty-six of these samples were attempted using DNA extracted from mosquito legs using the protocol described in Section 2.5, and 18 samples were attempted using DNA extracted from the mosquito abdomen using the protocol described in Section 2.6. The remaining samples were not subjected to COI barcoding because they were either easily identified morphologically or were morphologically identified similarly to the other submitted samples. Sequences were generated for 24 samples with lengths ranging from 267 to 619 bp; however, only five mosquito specimens could be identified to species with these sequences, including *Cx. tritaeniorhynchus* (N = 2), *Cx. gelidus* (N = 2), and *Ae. albopictus* (N = 1). Four samples were identified to genus, *Culex* (N = 1) and *Anopheles* (N = 1), tribe, Aedini (N = 1), or family, Culicidae (N = 1) (Supplementary file SF1). The remaining sequences had BLAST matches to multiple genera with ≥90% sequence identity, indicating an unresolved molecular identification. 

### 3.3. Mosquito–Host Networks by Land Cover Type

**Figure 2:**
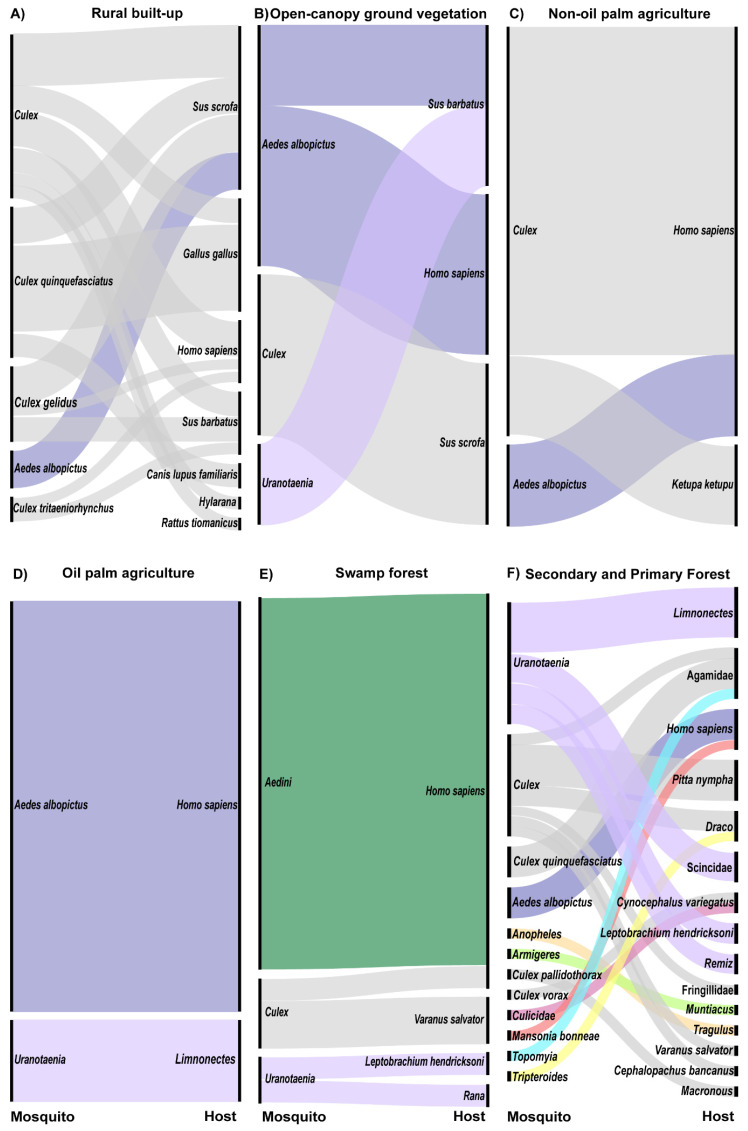
Alluvial plot showing mosquito and host networks at the land cover level for all land covers in which >5 bloodfed mosquitoes were collected (**A**) rural built-up (n = 36 mosquitoes), (**B**) open-canopy ground vegetation (n = 6), (**C**) non-oil palm agriculture (n = 6), (**D**) oil palm agriculture (n = 6), (**E**) swamp forests (n = 21), (**F**) secondary and primary forests (n = 36). Left nodes represent mosquito and right nodes represent host taxonomic groups. Colors of alluvial fans are consistent across figures and indicate mosquito genus.

Alluvial plots were created from those land covers with >5 mosquito–host interactions detected to visualize the relative frequency of connections between the sampled mosquitoes and hosts in the land cover types sampled. Barren, urban built-up, and rural built-up comprised three of the six human-modified land cover types in the study. Only one engorged mosquito, *Ae. albopictus*, was collected in barren land, and this mosquito had fed on a human. Four engorged *Culex quinquefasciatus* were collected in urban built-up land, three of which had fed on red jungle fowl and one on spotted dove. Within the rural built-up land cover type, seven host taxa were detected from 12 *Cx. quinquefasciatus*, six *Cx. gelidus*, two *Cx*. *tritaeniorhynchus*, 12 unidentified *Culex* and three *Ae. albopictus*. *Culex* species fed on mammals (70%; wild boar, humans, bearded pigs, dogs, and a Malaysian field rat), birds (27%, all red jungle fowl), and amphibians (3%, a golden-backed frog). Three *Ae. albopictus* in this land cover type fed on wild boar (Figure 2A).

Open-canopy ground vegetation, non-oil palm agriculture, and oil palm agriculture comprised the remaining three human-modified land cover types in the study. Three mammals were detected as hosts from six mosquitoes in the open-canopy ground vegetation land cover type. Bearded pig was identified as a host for one *Ae. albopictus* and one *Uranotaenia* sp., wild boar was identified as a host for two *Culex* sp., and humans were detected as a host from two *Ae. albopictus* (Figure 2B). Land under several agricultural crops, including cocoa (n = 1), durian (n = 1), fish (n = 1), and lime (n = 1) farms, made up the non-oil palm agriculture land cover type. In these agricultural lands, humans were identified from four *Culex* specimens and one *Ae. albopictus* (Figure 2C). Additionally, a buffy fish owl was identified as the host for one *Culex* sp. (Figure 2C). In oil palm plantations, humans were detected as hosts from five *Ae. albopictus* specimens and a forked-tongued frog from one *Uranotaenia* sp. (Figure 2D).

Three categories of forest were sampled in this study. In swamp forests, the most common mosquito collected was morphologically identified as *Verrallina,* but as molecular analyses to date have been inconclusive, these are here listed simply as Aedini. Humans were the only hosts detected from these 16 specimens (Figure 2E). Three *Culex* sp. from swamp forests fed on humans (33%) and water monitor lizards (67%) (Figure 2E). Amphibians were the only hosts detected from the *Uranotaenia* mosquitoes in this land cover type, including one spotted-litter frog and one brown frog (Figure 2E).

The majority of engorged mosquitoes were collected in secondary forests, while only 5 bloodmeals were identified from primary forest, thus these land cover types were combined into a single alluvial plot (Figure 2F). *Uranotaenia* was the most common genus from secondary forests; hosts for this genus of mosquitoes included reptiles (27%, skink), amphibians (64%, spotted litter frog and forked-tongued frog), and birds (9%, Eurasian penduline tit) (Figure 2F). *Culex* in secondary forests fed on birds (42%, pin-striped tit-babbler and fairy pitta), reptiles (50%, agamid lizard, flying lizard, and a water monitor), and mammals (8%, Malaysian colugo) (Figure 2F). Humans were detected as hosts for two *Ae. albopictus* specimens in secondary forests (Figure 2F). The remaining mosquito genera were collected as singleton samples and hosts encompassed a broad array of taxa (Figure 2F).

Primary forests yielded the only non-human primate bloodmeal, from a Horsfield’s tarsier, detected in this study. This bloodmeal was isolated from a single *Culex* specimen (Figure 2F). Unfortunately, COI barcoding results have not enabled a species-level identification of this specimen. *Culex* sp. also fed upon a finch and an agamid lizard in this land cover type (Figure 2F). A single *Ae. albopictus* collected in primary forests fed on human, and a single *Uranotaenia* mosquito fed upon a Eurasian penduline tit (Figure 2F).

### 3.4. Site Level Networks Between Mosquitoes and Hosts

For spillover of arboviruses to occur, vectors and hosts must be in close proximity. This may translate to a fine spatial scale, such as the site level, given the limited dispersal distances of most mosquitoes [56]. Therefore, sites were identified where either 1) a single mosquito taxonomic group was feeding on multiple host taxa or 2) two mosquito taxonomic groups were feeding on a single host taxon.

**Figure 3:**
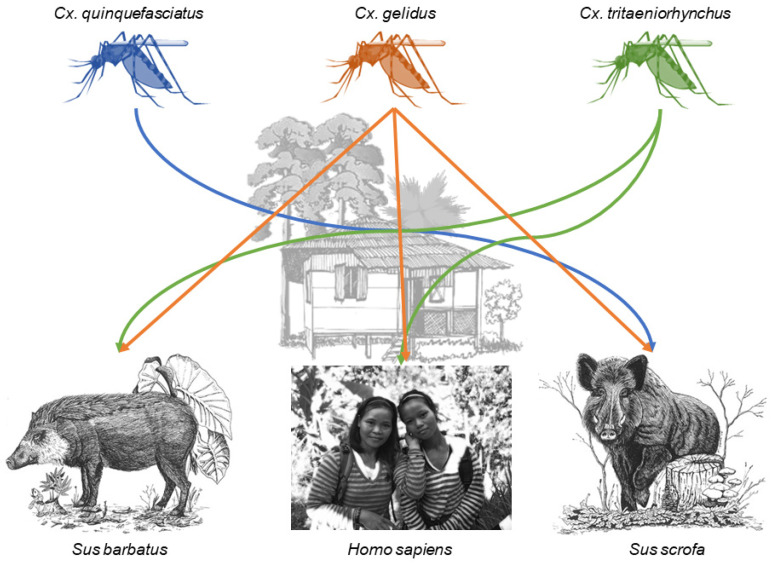
Connections between humans and pig species mediated by designated *Culex* species within the rural built-up site R_015. Samples sizes are found in Table 2. Host species images from left to right are Bornean bearded pig (*Sus barbatus*) [57]^†^, human (photograph taken by Katherine I. Young), and Eurasian wild boar (Sus scrofa) [58]^†^. † Licensee of the Licensed Material: Ecology, Conservation and Management of Wild Pigs and Peccaries, Bearded pig *Sus barbatus* (Müller, 1838), Eurasian wild boar *Sus scrofa* (Linnaeus, 1758), License Date: 03/26/2020, PLSclear Ref No.: 35570. Reproduced with permission of The Licensor through PLSclear

The first scenario, in which a single mosquito group fed on multiple taxa, played out at sites within the urban built-up (N = 1), rural built-up (N = 2), non-oil palm agriculture (N = 1), and secondary forest (N = 2) land cover types (Table 2). At site U_009, *Culex quinquefasciatus* fed on two avian species, red jungle fowl and spotted dove. Rural site R_015 had a network between four different *Culex* taxa and multiple hosts (Figure 3). One *Cx. gelidus* fed on humans, two on bearded pigs, and three on wild boar. Two *Cx. tritaeniorhynchus,* shared a similar feeding pattern as *Cx*. *gelidus*, feeding on humans and bearded pig. One *Cx. quinquefasciatus* fed on wild boar. At this site, three *Culex* spp. fed on humans, two on bearded pigs, four on wild boar, two on red jungle fowl, one on a *Hylarana* frog, and one on a Malaysian field rat. At site R_001, *Cx. quinquefasciatus* fed on both humans and wild boar. At site AG_017, a durian farm, two unidentified *Culex* sp. fed on a buffy fish owl and a human. At secondary forest site SF_017 two *Culex* sp. fed on fairy pitta birds and one on a flying lizard. Last, at secondary forest site F_123 two *Uranotaenia* mosquitoes fed on an amphibian, a spotted-litter frog, and a reptile, a skink.

The second scenario, in which multiple mosquito taxa feed on a single host species, only played out at one site within the rural-built up land cover type site R_013, where wild boar was host for one *Cx. quinquefasciatus* and two *Ae. albopictus* (Table 2). 

## 4. Discussion

Our data on mosquito bloodmeal sources from a range of land cover types in Sarawak, Malaysian Borneo demonstrate that bloodmeal analysis from mosquitoes can be used to identify a broad range of hosts in a biodiversity hot spot. In our sample of 134 engorged female mosquitoes, 87% of the bloodmeal sources were identified, similar to other studies of mosquito host feeding patterns [59,60,61,62]. Eighty-three percent of the 18 samples that failed to produce host identifications were collected in the KK and OP projects. Mosquitoes from these projects were not held on ice packs during transportation back to the laboratory, while samples from the LC project were held on ice, which may account for the greater success in amplifying samples from the LC project. Storage method and temperature has been shown to impact the success of DNA amplification from mosquito bloodmeals [63,64]. Further, we did not attempt to characterize the digestion status of the bloodmeals collected in any of the projects. DNA degradation of the bloodmeal occurs over time and negatively impacts the success of DNA extraction and bloodmeal identification [63,65,66]. It is also possible that certain host species were not detected due to the specificity of the primers used. A recent study on the Ryukyu Archipelago of Japan reported specialized feeding patterns of *Ae. baisasi* on 15 different fish species determined using universal COI primers and fish specific mitochondrial 12S rRNA primers [51].

Several mosquitoes known to act as key arbovirus vectors were analyzed in this study. Across seven land cover types, humans and boars were the only hosts detected from 16 *Ae. albopictus*, an important vector of human-endemic DENV within this region and a potential bridge vector for sylvatic DENV as well as other arboviruses. Despite its spread into urban areas [67], *Ae. albopictus* is thought to be sylvatic in nature and generalist in feeding behavior [68]. *Ae. albopictus* has been shown to feed on birds, amphibians, and reptiles across its global range, though the majority of bloodmeals characterized come from mammals [60,69]. Within its native range, *Ae. albopictus* seems to preferentially feed on humans, although the majority of studies supporting this conclusion have been conducted in urban and rural land cover types where humans are highly abundant [60,69,70]. However, Sivan et al. reported a reduction in human feeding by *Ae. albopictus* from urban to forested landscapes in India [71]. Our data revealed humans as hosts for *Ae. albopictus* in both secondary and primary forests where this species may play a role as a bridge vector in sylvatic DENV spillover to humans [7,33]. We detected no non-human primates as hosts for *Ae. albopictus*, although Khaklang and Kittayapong reported seven cases in which monkeys and humans were detected from a single bloodmeal from *Ae. albopictus* samples in sites near national parks in Thailand [72]. Further studies on the feeding behavior of *Ae. albopictus* in forests, would be valuable for better determining its role as an arbovirus bridge vector.

Several *Culex* species known to act as vectors of JEV were collected in this study, including *Cx. tritaeniorhynchus*, *Cx. gelidus*, and *Cx. quinquefasciatus* [73]. *Cx. tritaeniorhynchus* has been described as the main vector of JEV transmission in Southeast Asia, where it is highly zoophilic and facilitates amplification of the virus in pigs [6,73,74]. Humans and bearded pigs were the only hosts identified from *Cx. tritaeniorhynchus* from a single site in the rural built-up land cover type. The last study of JEV transmission and feeding habits of *Cx. tritaeniorhynchus* in this region of Sarawak was conducted in the early 1970s, and this species was also found to feed on pigs and humans in rural villages [75]. Based on the bloodmeals taken by other mosquito species, our data show a diversity of hosts available within the rural built-up land cover type; nonetheless, the 2 *Cx. tritaeniorhynchus* sampled selected only humans and pigs. The other JEV vectors mentioned above were also found to feed on Suidae, humans, and in the case of *Cx. quinquefasciatus*, birds, in urban and rural built-up land cover types. The majority of host feeding behavior studies for *Cx. quinquefasciatus* have been conducted in North America and Europe due to its high competence for West Nile virus (WNV) transmission. In this region, this species is an opportunistic feeder and bridge vector [76,77]. Hosts identified from *Cx. quinquefasciatus* across all land cover types were primarily birds, though none of the species documented are known hosts of JEV. *Cx. gelidus*, which previous studies have shown to be an opportunistic feeder [59,78], was only collected in the rural built-up land cover type where it fed on humans and Suidae. Importantly, all engorged *Cx. gelidus* and *Cx. tritaeniorhynchus* were collected from the same site, R_015, where their intertwined host utilization reveals potential risk for JEV transmission. The remaining *Culex* specimens were mostly identified only to genus but showed a wide land cover distribution and breadth of hosts, including the only non-human primate detected in this study, the Horsfield’s tarsier. This group of *Culex* also created within-site host networks, the most important of these with *Cx. tritaeniorhynchus* and *Cx. gelidus*, which created connections among six taxa. Identifying these individual species will provide valuable information about their potential vector status, and work to this end is ongoing. We focus on these site-level interactions not to draw general inferences, which is not possible due to the small samples sizes, but rather to offer a model for how this type of data should be parsed to gain maximum insight.

Two other groups of mosquitoes, *Uranotaenia* and a group of 16 individuals identified to the Aedini tribe, made up a significant portion of the bloodmeals identified in this study. *Uranotaenia* species are not considered relevant vectors of human disease, although WNV has been isolated from *Ur. unguiculata* in Eastern Europe [79]. Little is known about this genus of mosquitoes, but some species have been shown to preferentially feed on birds, herptiles, and invertebrate, annelid hosts [61,79]. Some of these feeding preferences were corroborated in our data, extending existing knowledge of the feeding habits of this genus. Humans were the only hosts detected from the Aedini group across multiple swamp forest sites, making this group of individuals the only complete specialists identified in this study. These samples were originally morphologically identified as belonging to *Verrallina*, a genus within the Aedini tribe forming a monophyletic group most closely related to *Aedes* [80]. The 95 known species of *Verrallina* primarily occur across the Indomalayan and Australasian biogeographic realms and prefer habitats with flushed pools of brackish water [81,82,83], similar to the swamp forest sites in which these Aedini specimens were collected. Though *Verrallina* is not considered a medically-important genus of mosquito vectors, they are considered pest species that frequently bite humans [82]. Further, some species have been shown to be experimentally competent for Barmah Forest and Ross River viruses in Australia [84,85]. Though these viruses are not known to occur in Borneo, solid identification of these mosquitoes is necessary to better understand their potential as vectors within this region.

The remaining mosquito taxa collected in this study represent single specimens from secondary forests. Of the five mosquitoes identified to genus, three genera, *Anopheles*, *Armigeres*, and *Mansonia*, include known vectors of human pathogens in this region [86]. Based on sample size, no clear host network information can be visualized for these species. However, the hosts detected from these specimens are diverse, two of which, Bornean bearded pig and fairy pitta, are considered vulnerable species by the International Union for Conservation of Nature (IUCN) [87]. Further, the only non-human primate detected in this study, Horsfield’s Tarsier, is also considered vulnerable by IUCN [87] and was a host for an unidentified *Culex* species. Bloodmeals from leeches have been used in Southeast Asia to detect mammalian hosts for conservation efforts [88], and Kocher et al. (2017) took a similar approach in French Guiana with dipteran vectors of disease [89]. This latter study sampled bloodfed Phlebotominae flies and mosquitoes across a gradient of anthropogenic pressure on the local native rainforest forest and compared the diversity of hosts identified. Similar to our data, they found that the diversity of hosts detected was reduced with the increase of anthropogenic disturbance of the surrounding forest [89]. Further, mosquito bloodmeals can be used to give insight into the prevalence of pathogens circulating in wildlife that can pose a significant disease risk and impact conservation efforts [90]. Given the impact of LCLUC on the biodiversity of terrestrial animals in Borneo [91,92,93] and diversity of hosts detected in our study, a targeted surveillance of mosquitoes and other hematophagous insects is warranted for use in species detections and conservation efforts.

Humans were detected as bloodmeal sources for mosquitoes, many of known vector status, in all land cover types investigated except, ironically, urban built-up. From these data we can conclude that humans are encountered and desired as hosts, despite a relatively high diversity of hosts available across all land cover types, and that there is potential for zoonotic arboviral spillover between land cover types. Future research should focus on arbovirus surveillance in mosquitoes in Sarawak using virus isolation and/or next generation sequencing from which host, vector, and pathogen can be detected. Such surveillance would be most profitable at the interior and border of agricultural, rural, and forested land cover types in Borneo where reservoir hosts occur and humans live in close proximity to reservoir species habitats [1,11,94].

Given that we identified 21 host taxa from a relatively small sample of mosquitoes, it is almost a certainty that more extensive sampling efforts would increase the richness of host species and vector species detected and the complexity of host–vector networks. However, the interactions identified in this study would still hold. Moreover, few studies have performed bloodmeal analyses from mosquitoes in Borneo; to date, only Brown et al. have reported the identification of 38 bloodmeals from mosquitoes in Sabah, Malaysian Borneo [52]. Thus, our data give valuable insight into the vector and host networks related to arbovirus transmission, especially JEV and DENV, which can be used for targeted surveillance of zoonotic arboviruses within this region of Borneo.

## Figures and Tables

**Table 1:** Description of hosts detected via cytochrome oxidase subunit I (COI) bloodmeal barcoding from specified mosquito genera or tribes in Sarawak. Whole numbers represent counts of host species detected. Percentages represent the proportion of bloodmeals detected from specified taxonomic groups for different mosquito genera.

Host	Common Name	*Aedes*	*Anopheles*	*Armigeres*	*Culex*	Culicidae	*Mansonia*	*Topomyia*	*Tripteroides*	*Uranotaenia*	Aedini
**Amphibian**					**1%**					**63%**	
*Hylarana spp.*	Golden-backed frog				1						
*Leptobrachium hendricksoni*	Spotted litter frog									3	
*Limnonectes spp.*	Forked-tongued frog									6	
*Rana spp.*	Brown frog									1	
**Avian**					**32%**					**13%**	
*Ketupa ketupu*	Buffy fish owl				1						
Fringillidae	Finches				1						
*Gallus gallus*	Red jungle fowl				12						
*Macronous spp.*	Pin-stripedtit-babbler				1						
*Pitta nympha*	Fairy pitta				4						
*Remiz spp.*	Eurasian pendulines									2	
*Streptopelia chinensis*	Spotted dove				1						
**Mammal**		**100%**	**100%**	**100%**	**52%**	**100%**	**100%**			**5%**	**100%**
*Canis lupus familiaris*	Domestic dog				2						
*Cephalopachus bancanus*	Horsfields tarsier				1						
*Cynocephalus variegatus*	Malayan colugo				1	1					
*Homo sapiens*	Human	12			10		1				16
*Muntiacus muntjak*	Barking deer			1							
*Rattus tiomanicus*	Malaysian field rat				1						
*Sus barbatus*	Bornean bearded pig	1			5					1	
*Sus scrofa*	Wild boar	3			12						
*Tragulus spp*	Mouse deer		1								
**Reptile**					**15%**			**100%**	**100%**	**19%**	
Agamidae	Dragon lizard				4			1			
*Draco spp.*	Flying lizard				2				1		
Scincidae	Skink									3	
*Varanus salvator*	Water monitor				3						
**TOTALS**		**16**	**1**	**1**	**62**	**1**	**1**	**1**	**1**	**16**	**16**

**Table 2:** Mosquito and host networks at the site level in sites where either a single mosquito taxon fed on multiple host taxa or multiple mosquito taxa fed on a single host taxon.

Site ID	Mosquito Taxa	Host Taxa	Number Bloodmeals Identified
Urban Built-Up			
U_009	*Culex quinquefasciatus*	*Gallus gallus*	1
	*Culex quinquefasciatus*	*Streptopelia chinensis*	1
**Rural Built-Up**			
R_015	*Culex gelidus*	*Homo sapiens*	1
	*Culex gelidus*	*Sus barbatus*	2
	*Culex gelidus*	*Sus scrofa*	3
	*Culex tritaeniorhynchus*	*Homo sapiens*	1
	*Culex tritaeniorhynchus*	*Sus barbatus*	1
	*Culex quinquefasciatus*	*Sus scrofa*	1
	*Culex species*	*Gallus gallus*	2
	*Culex species*	*Homo sapiens*	3
	*Culex species*	*Hylarana* species	1
	*Culex species*	*Rattus tiomanicus*	1
	*Culex species*	*Sus barbatus*	2
	*Culex species*	*Sus scrofa*	4
R_001	*Culex quinquefasciatus*	*Gallus gallus*	5
	*Culex quinquefasciatus*	*Sus scrofa*	1
R_013	*Aedes albopictus*	*Sus scrofa*	2
	*Culex quinquefasciatus*	*Sus scrofa*	1
**Durian Farm**			
Ag_017	*Culex species*	*Ketupa ketupu*	1
	*Culex species*	*Homo sapiens*	1
**Secondary Forest**			
SF_017	*Culex species*	*Draco* species	1
	*Culex species*	*Pitta nympha*	2
F_123	*Uranotaenia* species	*Leptobrachium hendricksoni*	1
	*Uranotaenia* species	*Scincidae* family	1

## Supplementary Materials

The following are available online at https://www.mdpi.com/2414-6366/5/2/51/s1, File S1: mosquito_bloodmeal_identifications.xlsx, File S2: failed_mosquito_amplicons.xlsx, Figure S1: Photographs of land cover types, Table S1: Land cover and site descriptions, Table S2: Bloodmeal PCR primers, Table S3: Bloodmeal PCR reaction information, Table S4: PCR reaction and conditions for cloning.
