# Supplementary material for: Identification of Mosquito Bloodmeals Collected in Diverse Habitats in Malaysian Borneo Using COI Barcoding
Source: Trop Med Infect Dis. 2020 Apr 1;5(2):51. doi: 10.3390/tropicalmed5020051 (PMC7344668; doi:10.3390/tropicalmed5020051)
Supplement: Supplementary file 1 [file tropicalmed-05-00051-s001.zip › tropicalmed-702383 proof-done supple/Supplementary-files_tropicalmed-702383.03122020/tropicalmed-702383 supplementary.docx]

Identification of Mosquito Bloodmeals Collected in Diverse Habitats in Malaysian Borneo Using COI Barcoding

Katherine I. Young ^1,^*, Joseph T. Medwid ^1^, Sasha R. Azar ^2,3,4^, Robert M. Huff ^5^, Hannah Drumm ^6^, Lark L. Coffey ^6,7^, R. Jason Pitts ^5^, Michaela Buenemann ^8^, Nikos Vasilakis ^2,3,9,10,11^, David Perera ^12^ and Kathryn A. Hanley ^1^

^1^ Department of Biology, New Mexico State University, Las Cruces NM 88003, USA; jtmedwid@nmsu.edu (J.T.M.); khanley@nmsu.edu (K.A.H.)

^2^ Department of Pathology, University of Texas Medical Branch, Galveston, TX 77555, USA; srazar@utmb.edu (S.R.A.); nivasila@utmb.edu (N.V.)

^3^ Department of Microbiology and Immunology, University of Texas Medical Branch, Galveston, TX 77555, USA

^4^ Institute for Translational Sciences, University of Texas Medical Branch, Galveston, TX 77555, USA

^5^ Department of Biology, Baylor University, Waco, TX 76706, USA; Robert_Huff1@baylor.edu (R.M.H.); Jason_Pitts@baylor.edu (R.J.P.)

^6^ School of Veterinary Medicine, University of California Davis, Davis, CA 95616, USA; hdrumm@ucdavis.edu (H.D.); lcoffey@ucdavis.edu (L.L.C.)

^7^ Department of Pathology, Microbiology & Immunology, University of California Davis, Davis, CA 95616, USA

^8^ Department of Geography, New Mexico State University, Las Cruces, NM 88003, USA; elabuen@nmsu.edu

^9^ Center for Biodefense and Emerging Infectious Diseases, University of Texas Medical Branch, Galveston, TX 77555, USA

^10^ Center for Tropical Diseases, University of Texas Medical Branch, Galveston, TX 77555, USA

^11^ Institute for Human Infection and Immunity, University of Texas Medical Branch, Galveston, TX 77555, USA

^12^ Institute of Health and Communiti Medicine, Universiti of Malaysia Sarawak, Sarawak 94300, Malaysia; dperera@unimas.my

***** Correspondence: kiy761@nmsu.edu

**
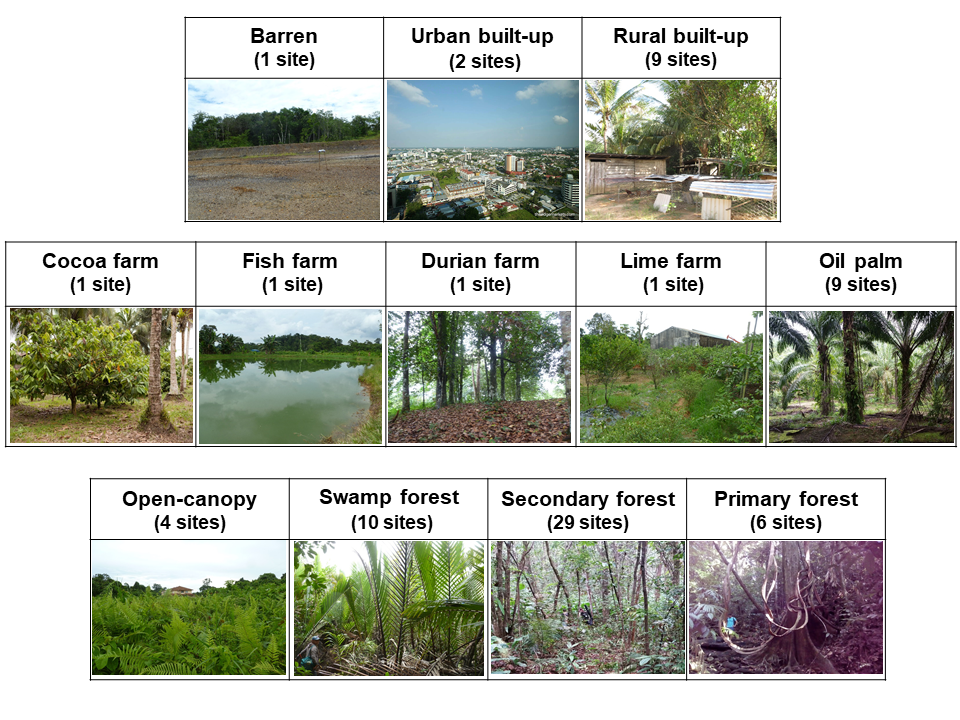
**

**Figure S1.** Photographs of one site each in the land cover types sampled in this study and the number of sites in which bloodfed mosquitoes were collected in each land cover type.

**Table S1.** Land cover and descriptions of sites in which bloodfed mosquitoes were collected. The number of bloodfed female mosquitoes is reported as well as the bloodmeals identified by COI barcoding. Refer to supplementary file SF1 for more detail.

| **Land Cover Type/Sites** | **Latitude** | **Longitude** | **Elevation(m)** | **Bloodfed Mosquitoes Collected (N)** | **Bloodmeals Identified (n)** |
| --- | --- | --- | --- | --- | --- |
| **BARREN: Denuded land cover with little to no vegetation cover** | | | | | |
| B_021 | 1.443117 | 110.37445 | 20 | 1 | 1 |
| **TOTAL** |  |  |  | **1** | **1** |
| **URBAN BUILT-UP: Highly urbanized area composed mostly of buildings and roads, vegetation restricted to small lawns.** | | | | | |
| U_009 | 1.558 | 110.3836 | 5 | 2 | 2 |
| U_016 | 1.419833 | 110.295133 | 28 | 2 | 2 |
| **TOTAL** |  |  |  | **4** | **4** |
| **RURAL BUILT-UP: Residential neighborhoods or villages, vegetation present in small yards; although, small-scale agricultural land is often in proximity** | | | | | |
| HOMMR | 1.279422 | 110.282778 | 46 | 1 | 1 |
| R_003 | 1.346567 | 110.412217 | 8 | 1 | 1 |
| PS_001 | 1.601533 | 110.33255 | 8 | 1 | 0 |
| R_006 | 1.50765 | 110.331717 | 12 | 1 | 1 |
| R_008 | 1.347467 | 110.4155 | 9 | 2 | 1 |
| R_001 | 1.396883 | 110.34145 | 20 | 6 | 6 |
| R_013 | 1.511817 | 110.373367 | 7 | 3 | 3 |
| R_015 | 1.47925 | 110.356383 | 15 | 22 | 22 |
| R_020 | 1.422533 | 110.4462 | 4 | 1 | 1 |
| **TOTAL** |  |  |  | **38** | **36** |
| **NON-OIL PALM AGRICULTURE: Vegetation consists of single crops and is present in defined plots or arranged in rows. Alternatively production of food animals occurs in defined plots** | | | | | |
| **Cocoa farm** | | | | | |
| FRMLR | 1.288008 | 110.281944 | 36 | 1 | 1 |
| **Fish farm** | | | | | |
| AG_011 | 1.431117 | 110.1714 | 27 | 1 | 1 |
| **Lime farm** | | | | | |
| AG_016 | 1.444767 | 110.3423 | 17 | 2 | 2 |
| **Durian farm** | | | | | |
| AG_017 | 1.406483 | 110.38025 | 23 | 2 | 2 |
| **TOTAL** |  |  |  | **6** | **6** |
| **OIL PALM PLANTATION: Large agricultural plantations with single crop matrix of oil palm trees** | | | | | |
| PO_592 | 1.41685 | 110.098999 | 27 | 1 | 1 |
| PO_487 | 1.41543 | 110.097 | 42 | 1 | 0 |
| PO_606 | 1.16747 | 110.318001 | 144 | 1 | 0 |
| PO_630 | 1.34487 | 110.088997 | 84 | 1 | 1 |
| PO_593 | 1.43984 | 110.109001 | 19 | 1 | 1 |
| PO_584 | 1.31394 | 110.083 | 55 | 1 | 1 |
| PO_530 | 1.32759 | 110.060997 | 54 | 1 | 1 |
| PO_477 | 1.33018 | 110.067001 | 49 | 1 | 0 |
| PO_617 | 1.304 | 110.105003 | 67 | 1 | 1 |
| **TOTAL** |  |  |  | **9** | **6** |
| **OPEN-CANOPY GROUND VEGETATION: Open-canopy areas with densely packed vegetative ground cover.** | | | | | |
| G_005 | 1.406567 | 110.350133 | 28 | 1 | 1 |
| G_007 | 1.439733 | 110.327783 | 19 | 1 | 1 |
| G_008 | 1.465417 | 110.346933 | 8 | 2 | 2 |
| G_014 | 1.4863 | 110.361633 | 8 | 2 | 2 |
| **TOTAL** |  |  |  | **6** | **6** |
| **SWAMP FOREST: Permanently or seasonally inundated forests, thick layer of partially decayed vegetation on ground.** | | | | | |
| PS_002 | 1.473067 | 110.3634 | 9 | 1 | 1 |
| PS_003 | 1.485717 | 110.367533 | 11 | 5 | 5 |
| PS_005 | 1.616533 | 110.434717 | 5 | 1 | 1 |
| PS_007 | 1.6022 | 110.423683 | 10 | 4 | 4 |
| PS_009 | 1.53385 | 110.38275 | 9 | 1 | 1 |
| PS_015 | 1.500433 | 110.401317 | 7 | 1 | 1 |
| PS_016 | 1.5003 | 110.4172 | 15 | 1 | 1 |
| PS_017 | 1.66975 | 110.204383 | 4 | 1 | 1 |
| PS_012 | 1.4691 | 110.446517 | 11 | 3 | 3 |
| PS_020 | 1.53325 | 110.400283 | 11 | 3 | 3 |
| **TOTAL** |  |  |  | **21** | **21** |
| **SECONDARY FOREST: Forest regeneration after human disturbance, smaller trees spaced closely together, dense vegetative ground cover, typically single layered canopy, little to no large vines.** | | | | | |
| SF_001 | 1.363517 | 110.275167 | 46 | 3 | 3 |
| SF_008 | 1.409317 | 110.33275 | 31 | 2 | 2 |
| SF_011 | 1.47975 | 110.352 | 18 | 1 | 1 |
| SF_014 | 1.400833 | 110.151467 | 113 | 1 | 1 |
| SF_017 | 1.405667 | 110.374567 | 19 | 4 | 3 |
| F_86 | 1.39124 | 110.072998 | 41 | 2 | 1 |
| F_77 | 1.33901 | 110.085999 | 31 | 1 | 1 |
| F_76 | 1.35124 | 110.089996 | 65 | 1 | 1 |
| F_36 | 1.38865 | 110.071999 | 45 | 1 | 1 |
| F_343 | 1.15276 | 110.330002 | 76 | 1 | 1 |
| F_316 | 1.33081 | 110.121002 | 267 | 1 | 1 |
| F_306 | 1.15395 | 110.331001 | 118 | 3 | 3 |
| F_291 | 1.38784 | 110.073998 | 39 | 1 | 1 |
| F_29 | 1.41328 | 110.098999 | 32 | 1 | 1 |
| F_261 | 1.17486 | 110.278 | 108 | 1 | 0 |
| F_246 | 1.38105 | 110.085999 | 27 | 1 | 1 |
| F_236 | 1.33891 | 110.087997 | 50 | 1 | 1 |
| F_232 | 1.37612 | 110.089996 | 37 | 1 | 0 |
| F_210 | 1.16408 | 110.324997 | 128 | 3 | 0 |
| F_200 | 1.44035 | 110.105003 | 27 | 1 | 0 |
| F_193 | 1.40384 | 110.018997 | 41 | 1 | 1 |
| F_180 | 1.41261 | 110.099998 | 34 | 1 | 1 |
| F_175 | 1.33422 | 110.068001 | 54 | 1 | 1 |
| F_168 | 1.30293 | 110.099998 | 65 | 1 | 1 |
| F_161 | 1.16713 | 110.325996 | 136 | 1 | 0 |
| F_136 | 1.39169 | 110.071999 | 40 | 2 | 1 |
| F_13 | 1.32271 | 110.122002 | 55 | 1 | 1 |
| F_123 | 1.28915 | 110.099998 | 57 | 2 | 2 |
| F_115 | 1.31812 | 110.114998 | 74 | 1 | 0 |
| **TOTAL** |  |  |  | **42** | **31** |
| **PRIMARY FOREST: Little to no human disturbance, large trees, multilayered canopy, limited ground vegetation, often have large vines.** | | | | | |
| PF_014 | 1.40055 | 110.3635 | 35 | 1 | 1 |
| PF_016 | 1.359767 | 110.355083 | 47 | 1 | 1 |
| PF_017 | 1.420867 | 110.419383 | 29 | 2 | 2 |
| F_301 | 1.19834 | 110.272003 | 54 | 1 | 1 |
| F_212 | 1.19621 | 110.279999 | 51 | 1 | 0 |
| F_250 | 1.43957 | 110.120003 | 28 | 1 | 0 |
| **TOTAL** |  |  |  | **7** | **5** |
| **GRAND TOTALS** | |  |  | **134** | **116** |

| **Table S2. Amplicon descriptions and primers used in PCR protocols to detect bloodmeals from mosquitoes.** | | | | |
| --- | --- | --- | --- | --- |
| **PCR Reaction** | **Amplicon**  **Size (bp)** | **Host** | **Primer** | **Sequence** |
| **Theiman et al., 2012 Protocol** | | | | |
| tRNA | ~ 1900 | Mammal | MaTrpF1 | 5’ AGACCRAGRGCCTTCAAAGCYCT 3’ |
| tRNA | ~ 1900 | Mammal | MaSerR1 | 5’ BRGGRGGTTCGATTCCTTCCTT 3’ |
| tRNA | ~ 1900 | Avian | AvTrpF1 | 5’ GGCCTTCAAAGCCTTAAAYAAGAGTT 3’ |
| tRNA | ~ 1900 | Avian | AvSerR4 | 5’ RRGGWWCGAYTCCTTCCTTTCTT 3’ |
| tRNA | ~ 1900 | Amphibian/Reptile | AmTrpF1 | 5’ AGWCCAARRRCCTTCAAAGYY 3’ |
| tRNA | ~ 1900 | Amphibian/Reptile | AmSerR1 | 5’ CAARRRCCTTCAAAGYCYYMAG 3’ |
| tRNA | ~ 1900 | Amphibian/Reptile | AmSerR1 | 5’ CAARRRCCTTCAAAGYCYYMAG 3’ |
| tRNA | ~ 1900 | Amphibian/Reptile | AmSerR2 | 5’ ATRYGBYYGRCTTGAAAYYRG 3’ |
| COI | 658 |  | VF1 | 5’ TTCTCAACCAACCACAAAGACATTGG 3’ |
| COI | 658 |  | VF1d | 5’ TTCTCAACCAACCACAARGAYATYGG 3’ |
| COI | 658 |  | VF1i | 5’ TTCTCAACCAACCAIAAIGAIATIGG 3’ |
| COI | 658 |  | VR1 | 5’ TAGACTTCTGGGTGGCCAAAGAATCA 3’ |
| COI | 658 |  | VR1d | 5’ TAGACTTCTGGGTGGCCRAARAAYCA 3’ |
| COI | 658 |  | VR1i | 5’ TAGACTTCTGGGTGICCIAAIAAICA 3’ |
| **Townzen et al. 2008 protocol** | | | | |
| COIshort | 324 |  | COI_F | 5’ GCAGGAACAGGWTGAACCG 3’ |
| COIshort | 324 |  | COI_R | 5’ AATCAGAAYAGGTGTTGGTATAG 3’ |
| **Cloning Protocol** | | | | |
| TOPO TA | 250 |  | CloneF | 5’-GCAGTWAATTTATTAC-3’ |
| TOPO TA | 250 |  | CloneR | 5’- ACTTCWGGRTGWCCAAARAATCA -3’ |

| **Table S3. PCR reaction information for each protocol used in bloodmeal identification.** | | |
| --- | --- | --- |
| **PCR Protocol** | **Reagent** | **Volume** |
| tRNA | H_2_O | 8.4 µl |
|  | 10x Buffer | 2.5 µl |
|  | MgCl_2_ (25 µM) | 2.5 µl |
|  | dNTP mixture (10 mM) | 2.5 µl |
|  | tRNA primers (20 µM) | 0.75 µl each = 6 µl total |
|  | Amplitaq enzyme | .01 µl |
|  | Bloodmeal DNA | 3 µl |
| TOTAL |  | 25 µl |
| COI | H_2_O | 18.9 µl |
|  | 10x Buffer | 2.5 µl |
|  | dNTP mixture (10 mM) | 0.5 µl |
|  | Vfmix primers (20 µM) | 0.5 µl |
|  | VRmix primers (20 µM) | 0.5 µl |
|  | HotStarTaq plus enzyme | 0.1 µl |
|  | tRNA product | 2 µl |
| TOTAL |  | 25 µl |
| COIshort | H_2_O | 8.7 µl |
|  | 10x Buffer | 5 µl |
|  | MgCl_2_ (25 µM) | 5 µl |
|  | dNTP mixture (10 mM) | 1 µl |
|  | Forward primer | 1 µl |
|  | Reverse primer | 1 µl |
|  | enzyme | 0.3 µl |
|  | Bloodmeal DNA | 3 µl |
| TOTAL |  | 25 µl |

| **Table S4. Description of the PCR reaction and conditions used to amplify a ~250 base pair region of the COI gene from bloodmeal DNA for mosquito species identification.** | | |
| --- | --- | --- |
| **PCR Reaction Information** | | |
| **Reagent** | | **Volume** |
| H_2_O | | 5.5 µl |
| Forward primer | | 2.5 µl |
| Reverse primer | | 2.5 µl |
| 2x GoTaq Green PCR Master mix | | 12.5 µl |
| Bloodmeal DNA | | 2 µl |
| **TOTAL** | | **25 µl** |
| **PCR Conditions** | | |
| **Number of Cycles** | **Temperature** | **Time** |
| 1 | 95˚C | 5 min |
| 5 | 95˚C | 45 s |
|  | 48˚C | 60 s |
|  | 72˚C | 45 s |
| 30 | 95˚C | 45 s |
|  | 52˚C | 60 s |
|  | 72˚C | 45 s |
|  | 72˚C | 5 min |
